# Evaluating the SarQoL^®^ Questionnaire as a Screening Tool for Sarcopenia among Korean Older Adults

**DOI:** 10.3390/healthcare12192000

**Published:** 2024-10-07

**Authors:** Haneul Lee, Jiyoun Kim

**Affiliations:** 1 Department of Physical Therapy, Gachon University, Incheon 21936, Republic of Korea; leehaneul84@gachon.ac.kr; 2 Department of Exercise Rehabilitation, Gachon University, Incheon 21936, Republic of Korea

**Keywords:** geriatric assessment, sarcopenia, quality of life, sensitivity, specificity

## Abstract

Background/objectives: Sarcopenia, characterized by the progressive loss of muscle mass and strength, poses significant risks to physical health, leading to a reduced quality of life (QoL), increased disability, and higher mortality rates among older adults. Early detection and intervention are crucial to prevent the cascading effects of sarcopenia, including falls, fractures, and hospitalization. This study determined an optimal cut-off point of the SarQoL^®^ score that can serve as an effective screening tool among community-dwelling Korean older adults. Methods: The study involved 451 South Korean older adults, assessing the correlation between SarQoL^®^ scores and sarcopenia as defined by the Asian Working Group for Sarcopenia (AWGS) criteria. Participants completed the Korean version of the SarQoL questionnaire. Results: Findings revealed that individuals diagnosed with sarcopenia had significantly lower SarQoL^®^ scores compared to non-sarcopenic participants, with a cut-off score of ≤58.5 providing good diagnostic accuracy (AUC = 0.768, sensitivity = 69.3%, specificity = 75.2%). Conclusions: These results underscore the questionnaire’s reliability and validity in screening for sarcopenia-related QoL impairment and its potential utility as a clinical tool. Implementing the SarQoL^®^ in routine assessments could improve clinical outcomes by enabling earlier and more precise identification of sarcopenia.

## 1. Introduction

Sarcopenia, characterized by gradual and progressive loss of skeletal muscle mass and strength, is a critical concern in aging populations [1,2,3]. As a syndrome directly associated with physical frailty, disabilities, and increased mortality [4,5,6,7], understanding and quantifying its impact on the overall quality of life (QoL) is important for both clinical and therapeutic strategies [8]. The progressive nature of sarcopenia not only impairs physical capability but also affects the overall well-being and independence of older adults. It often leads to a cascade of negative health outcomes, including an increased risk of falls, fractures, and hospitalization, as well as a decline in mental health due to reduced social interactions and increased feelings of depression [9,10]. These results highlight the need for early identification and intervention to mitigate the adverse effects of sarcopenia on QoL.

To gain comprehensive insights into the QoL of older adults with sarcopenia, a thorough evaluation using a validated questionnaire is essential [11]. The SarQoL^®^ questionnaire, developed specifically for individuals with sarcopenia, assesses seven domains of health-related QoL: physical and mental health, locomotion, body composition, functionality, activities of daily living, leisure activities, and fears [12,13]. With its meticulous consideration of 55 items covering seven health-related QoL domains, the SarQoL^®^ questionnaire comprehensively assesses multiple aspects of QoL, including physical, mental, and social dimensions, thereby offering a holistic view of the impact of sarcopenia on individuals [13]. This detailed assessment enables the SarQoL^®^ to capture not only the physical limitations associated with sarcopenia but also its effects on mental health and social interactions, which are crucial aspects of overall QoL. For example, the questionnaire evaluates how physical limitations lead to reduced participation in daily activities and social isolation, and how these limitations contribute to increased anxiety or fear of falls, thereby providing a comprehensive view of sarcopenia’s impact on individuals [14]. Additionally, recent literature has highlighted the importance of QoL assessment in sarcopenia, indicating that a diminished QoL is a consistent outcome for those affected by this condition [8]. The SarQoL^®^ questionnaire has been validated in various languages, demonstrating its versatility and sensitivity in detecting QoL impairments specific to sarcopenic sufferers [12,13,15,16,17]. This broad applicability underscores the necessity of incorporating QoL measures into routine sarcopenia assessments to guide clinical management and intervention strategies [18].

Furthermore, the operational definitions of sarcopenia have evolved, incorporating not only muscle mass and strength but also muscle function, as recommended by leading gerontological societies [19]. These definitions emphasize the relevance of implementing precise and reliable tools, such as the SarQoL, to evaluate outcomes and interventions. However, although these tools are available, their implementation in diverse populations and settings often reveals variability in QoL outcomes, suggesting a need for continued research on threshold values and cutoff points that can effectively signal the presence of sarcopenia across different demographic groups [20].

The prevalence of sarcopenia in Korea is increasing along with the rapid aging of its population. Recent estimates project that the proportion of people aged 65 and older will reach 20.3% by 2025, compared to 15.7% in 2020 [21]. Research indicates that sarcopenia affects approximately 13.1% of community-dwelling older adults in Korea, indicating a significant public health concern [22]. However, the cultural and lifestyle factors unique to this population necessitate localized validation of screening tools such as the SarQoL^®^ questionnaire. Previous studies have validated the SarQoL-K and demonstrated its applicability in the Korean context [23]. Building on this foundation, the present study sought to further refine the use of the SarQoL^®^ questionnaire by identifying a specific cutoff point that can effectively identify individuals with sarcopenia among community-dwelling Korean older adults.

Thus, this study aimed to evaluate the association between the SarQoL overall score and the presence of sarcopenia and to determine a specific cut-off point for the SarQoL^®^ questionnaire that can serve as an effective screening tool based on the revised Asian Working Group for Sarcopenia (AWGS) consensus criteria among Korean older adults.

## 2. Materials and Methods

### 2.1. Ethical Consideration

This research is a cross-sectional cohort study that adhered to the principles stated in the Declaration of Helsinki and received ethical approval approved by the institutional review board of Gachon University (1044396-202204-HR-082-02). All the participants provided written informed consent and signed the consent form before participating in the study. Additionally, the present manuscript was prepared in accordance with the recent version of the Standards for Reporting Diagnostic Accuracy (STARD) guideline [24].

### 2.2. Participants

A convenience sample of older adults who visited community centers in metropolitan areas of South Korea from February to June 2023 was used. Of the 664 adults aged 65 years and older, 496 completed the SarQoL^®^ questionnaire. Individuals with a body mass index (BMI) greater than 30 kg/m², those for whom calculating the appendicular skeletal muscle mass (ASM) was not appropriate, and those who had difficulties following the measurement instructions were excluded. Consequently, 451 older adults were included in the final analysis.

### 2.3. Measurements

The Inbody 120 (Inbody Corp., Seoul, Korea) was used to measure weight and skeletal muscle mass. ASM was then calculated using the following equation: ASM (kg) = 0.244 × body weight (kg) + 7.8 × height (m) + 6.6 × gender (1 for men and 0 for women) − 0.098 × age (years) + race (0 for whites, 1.4 for blacks, and −1.2 for Asians) − 3.3 [25]. Handgrip strength was measured using a Smedley-type handheld dynamometer (Fabrication Enterprises Inc., Elmsford, NY, USA). Participants were instructed to exert maximal effort for 5 s with both dominant and non-dominant hands, and two trials were performed, with the best performance recorded for analysis. Calf circumference was measured using a non-elastic tape measure with the participant seated and the measurement taken at the widest part of the calf. The short physical performance battery (SPPB) protocol was employed to assess physical performance, comprising three domains: balance, gait speed, and chair stand tests [26]. Based on the collected data, sarcopenia was diagnosed according to the AWGS criteria, considering participants with low muscle mass (ASM/ht^2^ < 7.0 kg/m^2^ for men and <5.7 kg/m^2^ for women) and low muscle strength (grip strength < 28 kg for men and <18 kg for women), and/or low physical performance (SPPB < 9). The AWGS criteria were used as the reference standard [27] in this study because of their status as the current consensus criteria and their applicability to samples recruited in South Korea.

The paper-based Korean version of the SarQoL^®^ questionnaire was used as the index test. The positivity of the index tests and cutoff points were defined using the receiver operating characteristic (ROC) curve. The Korean version of the SarQoL^®^ questionnaire comprises 22 questions, which include a total of 53 items, as some of the questions are structured in a matrix format. The SarQoL^®^ questionnaire generates an overall score derived from seven distinct domain scores: D1 (physical and mental health), D2 (locomotion), D3 (body composition), D4 (functionality), D5 (activities of daily living), D6 (leisure activities), and D7 (fears). Higher scores indicate better QoL. This version of the SarQoL^®^ questionnaire was used to assess an overall QoL, which was scored from 0 to 100 points based on the questionnaire responses [23,28]. The overall QoL score was calculated using a specialized MS Access database developed specifically for this purpose and can be obtained upon request. The sheet can be requested by contacting the team through the official website, www.sarqol.org “https://www.sarqol.org (accessed on 3 July 2023)”. A lower score indicates lower QoL and, consequently, a greater likelihood of sarcopenia-related disabilities [28]. The SarQoL^®^ questionnaire primarily evaluates the QoL impact of sarcopenia across multiple domains, capturing the broader effects of the condition on daily living and well-being. In contrast, the SARC-F questionnaire is designed as a brief screening tool to identify individuals at risk for sarcopenia by assessing physical function, such as strength and mobility. This study utilized both tools to compare their effectiveness in evaluating sarcopenia, recognizing that while SarQoL^®^ provides a detailed assessment of QoL, SARC-F serves as a quick screening method for clinical settings. To compare the performance of these two tools, the SARC-F was administered alongside theSarQoL^®^ questionnaire [29]. The SARC-F consists of 5 questions that assess strength, assistance in walking, rising from a chair, climbing stairs, and falling. Each item is scored from 0 to 2, resulting in a total score ranging from 0 to 10, where a score of ≥4 suggests the need for further examination for sarcopenia. SARC-F was developed to detect sarcopenia, and its performance has been widely evaluated [29].

The reference and index tests were completed by the participant during a single visit.

### 2.4. Statistical Analysis

Statistical analyses were performed using Statistical Product and Service Solutions (IBM Corp., Armonk, NY, USA), version 22.0, for Windows and MedCalculator software version 23.0.1 (MedCalc Software Ltd., Ostend, Belgium). The Kolmogorov–Smirnov test was used to test the normal distribution of the collected data. Binary logistic regression was used to evaluate the predictive probability of the SarQoL for sarcopenia diagnosis. Pearson’s chi-square test for cross-tabulation was conducted to assess the sensitivity and specificity. The receiver operating characteristic (ROC) curve was plotted with sensitivity on the vertical axis and 100-specificity on the horizontal axis. The optimal cutoff point for the overall SarQoL score was determined using the Youden index [30]. The area under the curve (AUC), representing diagnostic accuracy, was calculated using ROC curve analysis. An AUC value greater than 0.9 indicates high accuracy, 0.7–0.9 moderate accuracy, 0.5–0.7 low accuracy, and 0.5 a chance result [31]. Additionally, the DeLong method was used to compare the diagnostic abilities of the SarQoL^®^ questionnaire and the SARC-F questionnaire. An independent *t*-test was conducted to compare sarcopenia diagnostic indicators and SarQoL scores between the groups. Statistical significance was set at 0.05.

## 3. Results

All participants were assessed for sarcopenia using the AWGS criteria, and 125 (27.7%) of them were diagnosed with sarcopenia. The sarcopenic participants were older than those not diagnosed as sarcopenic (82.4 ± 6.0 vs. 75.8 ± 6.1 years, *p* < 0.001). The general characteristics of the study participants are presented in Table 1.

Significant positive correlations were found between the SarQoL and sarcopenia diagnostic indicators, including ASM (r = 0.272, *p* < 0.001), grip strength (r = 0.503, *p* < 0.001), and SPPB (r = 0.546, *p* < 0.001). Binary logistic regression showed that SarQoL score was significantly associated with sarcopenia (OR: 0.937; 95% CI: 0.923–0.952), indicating that an increase of one unit in SarQoL score decreased the probability of sarcopenia by approximately 6.5%.

The cutoff point of the SarQoL overall score as a criterion for predicting sarcopenia had significant results (*p* < 0.001), with an AUC of 0.768 (95% CI: 0.715 to 0.817). The Youden index was maximized at ≤58.5 points for the overall SarQoL score (Jc = 0.445, Se = 0.693, and Sp = 0.752), indicating good accuracy. Table 2 presents the index test results of the reference standards for the diagnosis of sarcopenia. A significant relationship was observed between the index and reference standard tests (*p* < 0.001). The AUC for the SARC-F was 0.794 (95% CI: 0.748 to 0.835).

**Figure 1:**
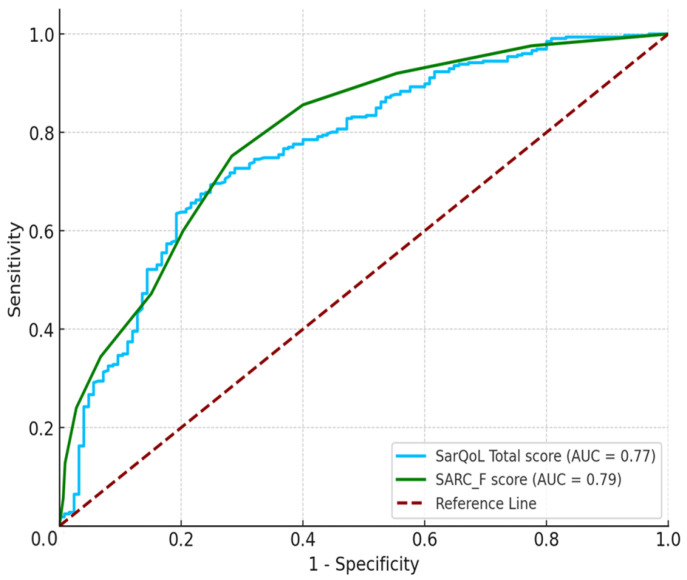
Comparison of ROC curves of the SarQoL^®^ score with the SARC-F score.

Table 3 describes the differences between the diagnostic abilities of the SarQoL (AUC = 0.768) and SARC-F (AUC = 0.794). No significant difference was found in AUC values between the SarQoL and SARC-F (*p* > 0.05) (Figure 1).

Table 4 illustrates the differences in the SarQoL scores between participants with and without sarcopenia. Participants diagnosed with sarcopenia had significantly lower overall SarQoL scores (50.5 ± 15.8) compared to those without sarcopenia (66.5 ± 15.4), with a *p*-value of <0.001. This trend was consistent across all seven domains of the SarQoL^®^ questionnaire. For instance, in the “Physical and Mental Health” domain, sarcopenic participants scored 48.1 ± 16.7, significantly lower than the 60.5 ± 17.2 score of non-sarcopenic participants. Similarly, in the “Locomotion” domain, sarcopenic participants scored 45.2 ± 21.9, compared to 61.9 ± 23.4 in those without sarcopenia. Other domains such as “Body Composition,” “Functionality,” “Activities of Daily Living”, “Leisure Activities”, and “Fears” all displayed statistically significant lower scores in sarcopenic participants, underscoring the profound impact of sarcopenia on QoL across various aspects.

## 4. Discussion

This study substantiated the validity and reliability of the SarQoL^®^ questionnaire as an essential tool for assessing QoL among Korean community-dwelling older adults with sarcopenia. Consistent with prior research [18,32,33] our findings underscore the significant correlation between sarcopenia severity and diminished QoL, thereby reinforcing the need for specialized tools such as the SarQoL in clinical settings.

The SarQoL^®^ questionnaire, with a cut-off value of ≤58.5, demonstrated good diagnostic accuracy, as evidenced by an AUC of 0.768, offering a sensitivity of 69.3% and specificity of 75.2%. Although this falls short of the 80% threshold often used to define excellent diagnostic tools, it remains a valuable screening tool. This finding aligns with those of previous studies, such as Beaudart et al. and Geerinck et al., who also reported a strong discriminative ability of the SarQoL score in identifying sarcopenic individuals [18,33]. For instance, Geerinck et al. identified a cut-off point of ≤52.4 in a European cohort, resulting in an AUC of 0.771, sensitivity of 64.7%, and specificity of 80.5%, similar to our findings, albeit with slight variations likely because of population differences [33].

In this study, the SarQoL^®^ was compared with the SARC-F, a well-known sarcopenia screening tool. Although the SARC-F displayed a slightly higher AUC (0.794) than the SarQoL^®^, the difference was not statistically significant, suggesting that both tools were comparably effective in detecting sarcopenia in this population. However, SarQoL^®^ offers additional value by assessing the broader impact of sarcopenia on QoL, which SARC-F does not capture. This distinction between SarQoL^®^ and SARC-F highlights their complementary role: while SARC-F is effective in identifying physical symptoms, SarQoL^®^ provides insight into the broader psychosocial and functional consequences of sarcopenia. This makes the SarQoL^®^ a more comprehensive tool, particularly valuable in contexts in which understanding the overall well-being of individuals with sarcopenia is crucial.

A key strength of this study lies in establishing a cut-off value tailored specifically to the Korean older adult population, marking the first such effort in an Asian cohort. Although previous research has primarily focused on European populations [33], our study reflects the unique cultural and lifestyle factors of Korean older adults. A validation study by Yoo et al. also confirmed the robust psychometric properties of the SarQoL-K, supporting its applicability across different cultural contexts. By providing a population-specific threshold, our study enhances the clinical utility of SarQoL^®^ in Korea and underscores the importance of considering demographic and cultural differences in clinical research.

The strong association between the SarQoL scores and sarcopenia severity highlights the multifaceted impact of sarcopenia in older adults. Sarcopenia, characterized by the loss of muscle mass and strength, directly impairs physical mobility, leading to reduced independence and diminished mental well-being [34,35]. The resultant psychosocial effects, such as decreased social interactions and heightened depression, further contribute to the lower QoL observed in sarcopenic individuals [36]. These findings underscore the importance of addressing both physical and psychological aspects of sarcopenia in clinical practice. The SarQoL^®^ questionnaire’s holistic approach, which includes mental and social dimensions alongside physical health, aligns with the shift toward patient-centered care in geriatric medicine [13]. Early identification of sarcopenia using the SarQoL can facilitate timely interventions that not only aim to preserve muscle function but also enhance overall QoL [13]. This is particularly crucial, as sarcopenia often leads to a cascade of health declines, including an increased risk of falls, fractures, and hospitalization. Therefore, integrating SarQoL assessments into routine screening could play a pivotal role in improving the health outcomes of older adults.

Given the increasing prevalence of sarcopenia among the aging population, this research is crucial for enabling early and accurate identification and intervention [19]. Although traditional diagnostic methods focus on physical metrics such as muscle mass and strength, the SarQoL^®^ questionnaire offers a more holistic perspective by integrating mental and social aspects with physical health [28]. By establishing a specific cutoff score, our study introduces a practical tool that can be seamlessly integrated into clinical practice to enhance early screening and management of sarcopenia. Early detection is critical, as timely interventions can prevent further decline in muscle function and QoL, thereby reducing the risk of associated disabilities. The consistently lower SarQoL^®^ scores across multiple domains in participants with sarcopenia demonstrated the questionnaire’s ability to capture the broad-ranging effects of sarcopenia, offering insights beyond traditional physical assessments.

Despite its strengths, this study has several limitations. First, the cross-sectional design limits our ability to infer causality between low SarQoL^®^ scores and the presence of sarcopenia. Longitudinal studies, such as prospective cohort studies, would be beneficial to track changes in QoL and sarcopenia status over time. Second, instead of directly measuring ASM, it was estimated using a formula based on body weight, height, age, and gender, which may impact the accuracy of sarcopenia diagnosis. Although participant characteristics, such as comorbidities and lifestyle factors, were considered and reported, the lack of detailed clinical examinations before body composition analysis could have resulted in undetected conditions influencing the results. Third, the convenience sampling method limits generalizability, particularly for rural or culturally different populations. Moreover, as the study used AWGS criteria, the findings may not be comparable to those using other sarcopenia definitions. Lastly, the SarQoL^®^ cut-off had sensitivity (69.3%) and specificity (75.2%), indicating its utility as a screening tool, though it may miss some cases, highlighting the need for supplementary diagnostic measures.

In summary, this study highlights the importance of culturally tailored tools in clinical practice and suggests that the SarQoL^®^ questionnaire, with its cut-off, can play a pivotal role in the early screening and management of sarcopenia in Korean older adult populations. Given the clinical significance of the SarQoL^®^ cut-off value identified in this study, its integration into routine clinical practice could facilitate more effective screening and management of sarcopenia in older adults. This cut-off provides a practical reference for clinicians to stratify patients based on their risk of sarcopenia-related complications, enabling early intervention and personalized treatment plans aimed at improving overall QoL. Additionally, the SarQoL^®^ cut-off complements existing tools like SARC-F, with SarQoL^®^ offering insight into QoL and SARC-F focusing on physical symptoms. Used together, these tools can provide a more comprehensive assessment of sarcopenia and guide further diagnostic testing and interventions. Future research should focus on validating this cut-off in diverse populations and exploring its utility in combination with other screening tools, such as SARC-F, to enhance diagnostic accuracy and optimize patient care. Such efforts would further solidify the role of SarQoL^®^ as a comprehensive tool for both diagnostic and therapeutic decision-making in the context of sarcopenia.

## 5. Conclusions

This study advances the field of sarcopenia research by providing a validated tool that enhances early screening and comprehensive assessment of sarcopenia. The SarQoL^®^ questionnaire, with its established cut-off point, has the potential to significantly improve clinical outcomes by facilitating earlier and more accurate identification of sarcopenia, leading to timely, targeted interventions. Future research should continue to validate and expand these findings to ensure that the SarQoL^®^ questionnaire becomes an integral part of sarcopenia management across diverse global populations.

## Figures and Tables

**Table 1:** General characteristics of the participants (N = 451).

Characteristics	No Sarcopenia (*n* = 326)	Sarcopenia (*n* = 125)	*p*-Value
Age (years)	75.8 ± 6.1	82.4 ± 6.0	<0.001
Sex			
Women	223 (68.4)	116 (92.8)	<0.001
Men			
BMI (kg/m^2^)	24.7 ± 2.7	22.7 ± 2.8	<0.001
ASM (kg/m^2^)	6.9 ± 1.3	5.2 ± 0.9	<0.001
Skeletal muscle mass (kg)	21.8 ± 3.9	17.3 ± 2.8	<0.001
Calf circumference (cm)	34.5 ± 2.7	31.0 ± 2.3	<0.001
Grip strength (kg)	23.8 ± 6.6	14.5 ± 4.4	<0.001
SPPB (score)	10.6 ± 1.9	7.2 ± 3.0	<0.001
Education level			
~6 years	76 (23.3)	67 (53.6)	<0.001
7~ years	250 (76.7)	58 (46.4)	
Living alone			
Yes	125 (38.3)	58 (46.4)	0.134
No	201 (61.7)	67 (53.6)	
Smoking			
Yes	53 (16.3)	6 (4.8)	0.001
No	273 (83.7)	119 (95.2)	
Regular physical activity			
Yes	274 (84.3)	87 (70.2)	0.001
No	51 (15.7)	37 (29.8)	
Comorbidities (yes)			
Diabetes	79 (25.7)	38 (33.0)	0.144
Hypertension	169 (54.7)	67 (57.8)	0.586
Hyperlipidemia	118 (38.2)	28 (24.1)	0.008

Abbreviations: BMI, body mass index; ASM, appendicular skeletal muscle mass; SPPB, short physical performance battery. Note: Values are expressed as mean ± SD or n (%).

**Table 2:** Cross-tabulation of the index test with the reference standards.

		Index Test		
		Sarcopenia Negative	Sarcopenia Positive	Total
Reference Standards	Sarcopenia Negative	225 (67.9%)	31 (12.1%)	256 (100%)
	Sarcopenia Positive	101 (51.8%)	94 (48.2%)	195 (100%)
	Total	326 (72.3%)	125 (27.7%)	451 (100%)
χ2 (*p*)	71.984 (*p* < 0.001)			

**Table 3:** Comparison of AUC values between the ROC curve of SarQoL and that of SARC-F.

	AUC	95% CI	*p*-Value
SarQoL	0.768	0.715–0.817	
SARC-F	0.794	0.748–0.835	0.441 ^a^

Abbreviations: AUC, area under the curve; CI, confidence interval; ^a^: comparison of AUC between the ROC curve of SarQoL and SARC-F.

**Table 4:** Sarcopenic QoL of the participants (N = 451).

Characteristics	No Sarcopenia (*n* = 326)	Sarcopenia (*n* = 125)	*p*-Value
SarQoL Total score	66.5 ± 15.4	50.5 ± 15.8	<0.001
D1 Physical and mental health	60.5 ± 17.2	48.1 ± 16.7	<0.001
D2 Locomotion	61.9 ± 23.4	45.2 ± 21.9	<0.001
D3 Body composition	62.4 ± 18.2	50.0 ± 16.8	<0.001
D4 Functionality	72.6 ± 16.7	57.7 ± 17.8	<0.001
D5 Activities of daily living	65.8 ± 19.6	44.5 ± 20.9	<0.001
D6 Leisure activities	57.3 ± 25.6	47.5 ± 25.4	<0.001
D7 Fears	88.7 ± 10.5	83.6 ± 12.4	<0.001

Note: All values are expressed as mean ± SD (scores).

## Data Availability

The datasets generated in this study are available from the corresponding author upon request.
